# Non-Invasive Multiparametric Approach To Determine Sweat–Blood Lactate Bioequivalence

**DOI:** 10.1021/acssensors.2c02614

**Published:** 2023-04-08

**Authors:** Genis Rabost-Garcia, Valeria Colmena, Javier Aguilar-Torán, Joan Vieyra Galí, Jaime Punter-Villagrasa, Jasmina Casals-Terré, Pere Miribel-Catala, Xavier Muñoz, Joan Cadefau, Josep Padullés, Daniel Brotons Cuixart

**Affiliations:** † Department of Mechanical Engineering (DEM), Universitat Politècnica de Catalunya-BarcelonaTech (UPC), 08222 Terrassa, Spain; ‡ Onalabs Inno-hub S.L., 08290 Cerdanyola del Vallès, Spain; § Electronics and Biomedical Engineering Department, Universitat de Barcelona (UB), 08028 Barcelona, Spain; ∥ Institut Nacional d’Educació Física de Catalunya (INEFC), 08038 Barcelona, Spain; ⊥ Associació Chronojump, 08013 Barcelona, Spain; # Consell Català de l’Esport, 08950 Esplugues de Llobregat, Spain

**Keywords:** wearable sensors, sweat analysis, lactate monitoring, sport, multiparametric, machine learning

## Abstract

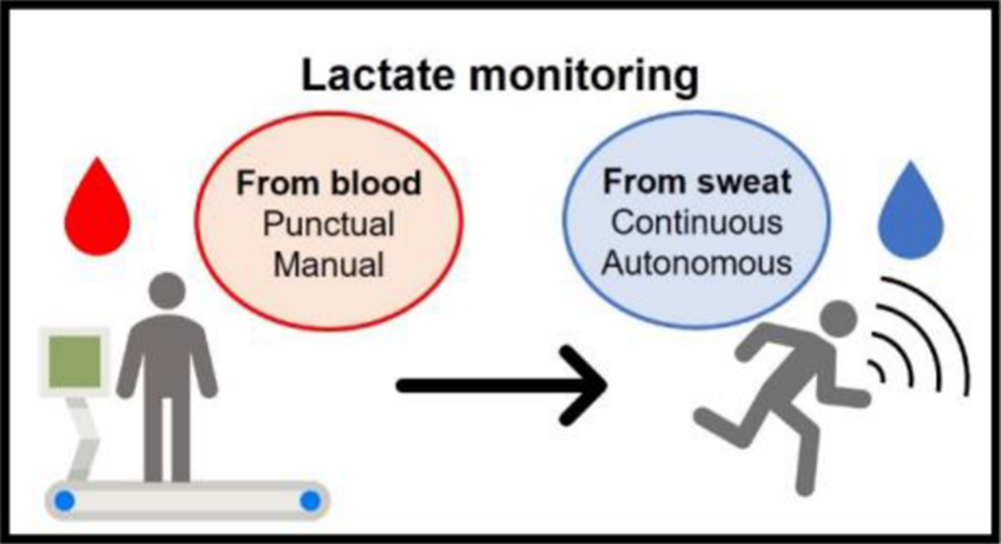

Many sweat-based wearable monitoring systems have been recently proposed, but the data provided by those systems often lack a reliable and meaningful relation to standardized blood values. One clear example is lactate, a relevant biomarker for both sports and health sectors, with a complex sweat–blood bioequivalence. This limitation decreases its individual significance as a sweat-based biomarker. Taking into account the insights of previous studies, a multiparametric methodology has been proposed to predict blood lactate from non-invasive independent sensors: sweat lactate, sweat rate, and heart rate. The bioequivalence study was performed with a large set of volunteers (>30 subjects) in collaboration with sports institutions (Institut Nacional d’Educació Física de Catalunya, INEFC, and Centre d’Alt Rendiment, CAR, located in Spain). A neural network algorithm was used to predict blood lactate values from the sensor data and subject metadata. The developed methodology reliably and accurately predicted blood lactate absolute values, only adding 0.3 mM of accumulated error when compared to portable blood lactate meters, the current gold standard for sports clinicians. The approach proposed in this work, along with an integrated platform for sweat monitoring, will have a strong impact on the sports and health fields as an autonomous, real-time, and continuous monitoring tool.

Nowadays, sweat is one of the most preferred body fluids for non-invasive continuous monitoring, due to its comfortable access and wide source of relevant biomarkers such as electrolytes and metabolites.^1^ However, sweat analysis implies a set of challenges: irregular sampling, contamination from skin and with old samples, evaporation, and low-volume analysis.^2^ In recent years, technological efforts have been carried out to solve these issues by the use of microfluidics for proper sampling,^3−5^ miniaturized sensors for low-volume analysis,^6−9^ and flexible electronics for wearable integration.^10,11^ Therefore, a great number of sweat wearable devices have been proposed, which can provide continuous and remote monitoring of multiple biomarkers of interest.^12^

One key challenge under-addressed in most sweat-based monitoring systems is the need to stablish a reliable bioequivalence to blood, the current gold standard for biochemical information. This issue is critical for providing meaningful information and to increase acceptance among stakeholders. However, the pathway from plasma to sweat is greatly dependent on the molecule nature, and some of the mechanisms involved remain still unknown.^13^ Specific models have been proposed to describe this transport in order to provide a reference framework to stablish this relation.^14^ For some biomarkers, such as ethanol, their small size and lipophilic nature result in a 1:1 ratio between sweat and blood levels, making them an ideal candidate for sweat monitoring, as demonstrated by Hauke et al.^15^ For other biomarkers, the pathway is not as direct as with ethanol: glucose presents lower concentrations in sweat compared to blood (up to 100-fold),^16^ but several studies support a correlation between them.^17,18^

Lactate is a small polar molecule end product of the glycolysis pathway related to anaerobic physical activity, which showed a poor correlation between sweat and blood levels^19^ due to an unclear path from plasma and the interference of the lactate secreted by the sweat gland itself. Therefore, stakeholders are reluctant to use it for blood lactate prediction.^20^ Some studies did find a relationship with the exercise intensity when accounting for the sweat rate, using the lactate excretion rate (LER)^21^ or even with blood levels when assessing variation rates instead of absolute values, avoiding the misleading effect of lactate produced by the sweat gland.^22,23^ The large interest in a non-invasive lactate monitoring system along with the new generation of wearable devices produced a new set of studies,^24−28^ learning from the limitations of the pre-wearable era regarding sampling and sweat rate control.^29^ Recently, Seki et al.^30^ were capable of detecting the lactate threshold from sweat measurements and showing a significant correlation with both blood lactate and ventilatory thresholds. Therefore, lactate analysis from sweat seems possible, but more effort is needed to provide a reliable bioequivalence. Wearable technology must take advantage of all the knowledge produced so far in physiological-orientated studies combined with the new tools developed in recent years.

In this work, a multiparametric bioequivalence study is proposed in order to overcome the challenges associated with lactate monitoring and stablish a relationship between blood lactate and non-invasive parameters: sweat lactate, sweat rate, and heart rate. For sweat-based measurements, advanced microfluidic sampling methods were used with the objective to obtain reliable data. For the lactate sensor, a commercial lateral flow strip was used because of its robustness and milder storing conditions compared to self-developed sensors. For the sweat rate, the key to correct for the dilution of the excreted lactate, a volumetric microfluidic patch combined with colorimetric detection was developed based on the patch used by Baker et al.^31^ in extensive field tests. Data were gathered from 32 volunteers during different typologies of exercise (cycling and running) and with variated protocols (increasing or constant intensity load) in order to have a wide range of realistic scenarios. Then, a multiparametric approach was applied in order to obtain a model capable of predicting reference blood lactate levels using non-invasive data, along with basic subject metadata, with enough accuracy to provide a trustful, autonomous, and continuous tool to both athletes and sports clinicians.

## Experimental Section

### Materials

Dibasic sodium phosphate, monobasic sodium phosphate, sodium chloride (NaCl), potassium chloride (KCl), urea, d-glucose, ascorbic acid, l-(+)-lactic acid, ammonium chloride (NH4Cl), Whatman 50 filter paper, and erioglaucine disodium salt were obtained from Sigma Chemical Co. All solutions were prepared using distilled water. ARcare 90106, ARcare 90445, and ARflow 93049 pressure-sensitive adhesives used for the sampling patches were kindly provided by Adhesives Research. Polymethylmethacrylate, ethanol 96%, sterile gauzes, and cotton swabs were bought from local stores.

### Sweat Lactate Sensor

Lactate Pro-2 test strips (Akray, Kyoto, Japan) were used as a single-use sweat lactate sensor. In vitro characterization was carried out by capillary absorption of the sample, and a commercial potentiostat (Palmsens 4, Palmsens BV, Netherlands) was used for the electrochemical methods. For determining the optimal operating potential, cyclic voltammetry was applied to a 10 mM lactic acid solution in 0.1 M phosphate buffer at pH 6.3. The potential was scanned from −0.1 to 0.8 V at a scan rate of 0.02 V/s, repeated up to 15 cycles to check system stability. Chronoamperometry at increasing concentrations of lactic acid in artificial sweat was used to test the sensor response. The artificial sweat solution consisted of phosphate buffer (pH = 6.5), 50 mM NaCl, 0.17 mM glucose, 5 mM NH4Cl, 20 mM urea, 0.03 mM ascorbic acid, and increasing lactate concentrations (0.5, 4, 8, 12, 20, 30, 40, and 70 mM). The potential was fixed at 0.05 V, and the current was measured for 200 s. As the sensor is single-use, a different test strip was used for each measurement (*N* = 4 for each lactate concentration). Custom instrumentation was used to perform the chronoamperometric measurement and remotely communicate the results to a mobile app through Bluetooth in in vivo tests. The instrumentation architecture was based on a previously published potentiostat^32^ and encapsulated in a plastic housing for strip insertion.

### Sampling Patch for Sweat Lactate

A sampling patch was defined for the recollection and transport of sweat samples to be captured by the test strip. The patch was constructed using adhesives that were laser-cut (BCN3D, Ignis) and laminated manually using alignment pins. Patch dimensions were 44 × 44 mm. Detailed information about the construction can be found in the Supporting Information.

### Sweat Rate Sensor

The sweat rate sensor consists of a microfluidic channel fabricated by laser-cutting (BCN3D, Ignis) and manual lamination using alignment pins. The sweat rate sensor dimensions were 53 × 30 mm. A filter paper (Whatman 50) was soaked with blue dye (20 μL of 800 μM erioglaucine solution) and left to dry at room temperature. This filter paper was placed at the inlet for providing color to sweat and facilitate visual inspection of the sweat front along the channel. Geometric dimensions of the microfluidic channel were measured from a subset of devices in order to provide averaged values for the volume calculation. The width and length were measured using an optical microscope (AM4515ZT-Edge, Dino-Lite), while the height was measured using an optical interferometer (Profilm3D, Filmetrics). In vitro characterization was carried out by injecting DI water with a syringe pump (UMP3, WPI, USA) at a constant flow rate of 1 μL/min. The fluid front position was captured using a smartphone camera for both in vitro and in vivo tests. Detailed information about the construction and sweat rate measurement can be found in the Supporting Information.

### In Vivo Studies

**Figure 1:**
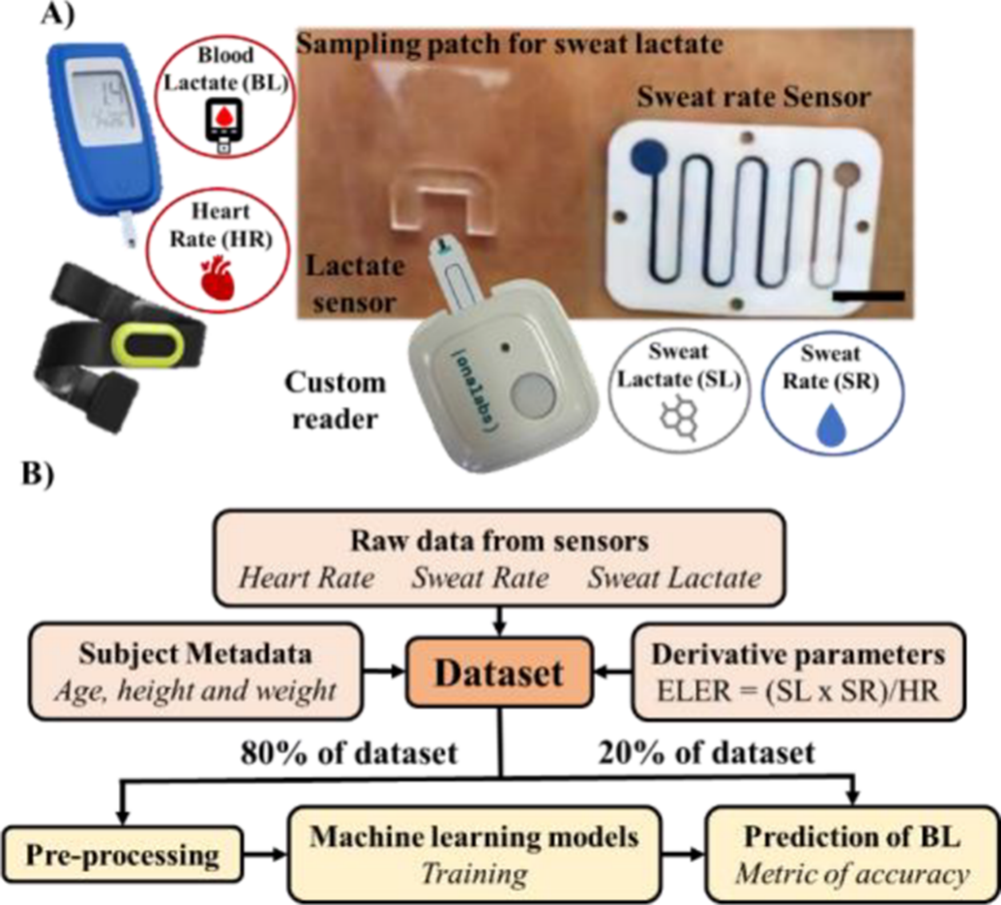
Methodology employed in the study. (A) Scheme of the different sensors used (blood lactate, heart rate, sweat lactate, and sweat rate). Scale bar = 1 cm. (B) Scheme of the data analysis pipeline followed in this work. ELER (exertion and lactate excretion rate) is the product of sweat lactate (SL) and sweat rate (SR) divided by the heart rate (HR).

The detailed protocol used for in vivo tests can be found in the Supporting Information. Briefly, skin was cleaned with ethanol, DI water, and dry sterile gauzes to avoid contamination and to ensure a good attachment of the adhesive patches. The patches and the heart rate monitor were placed at the chest area, and the physical test was started. When the subject started sweating, simultaneous measurements of blood lactate, heart rate, sweat lactate, and sweat rate were taken. Blood was extracted from earlobes, and the lactate measurement was carried out by commercial portable meters and the corresponding test strips (Lactate Pro2, Akray, Japan and Lactate Plus, Nova Biomedical, USA). For some subjects, a sample of blood was stored in a capillary for posterior analysis with colorimetric reference instrumentation (Diaglobal, Germany). For sweat lactate, the adapted test strip was inserted in the custom reader, which started sending real-time data of chronoamperometry to the mobile app. The sweat sample was captured from the sampling patch, making sure that after each measurement, the capture zone was cleaned. For the sweat rate measurement, a picture was taken of the microfluidic device with a smartphone to position the sweat front. This process was repeated for each set of measurements during the test with careful attention to time traceability. Figure 1A shows a scheme of all the sensors used during an in vivo test, and the procedure is described in Figure S6. A total of 32 subjects participated in the study (Table 1).

**Table 1:** Information of the Subjects of the Study

age [years]	21 ± 4
gender	21 males and 11 females
height [m]	1.74 ± 0.09
weight [kg]	66 ± 9
training level	20 amateurs and 12 athletes
skin condition	healthy, Caucasian (cleaned)

### Data Analysis

The data analysis was carried out using Excel (Microsoft) and R, and a general overview of the process is shown in Figure 1B. The analysis process started by extracting data from the sensors and constructing the dataset, combining all subjects with their corresponding metadata (subject information and environmental conditions) and sequential measurements. Besides, additional parameters were calculated from the initial measurements such as the exertion and lactate excretion rate (ELER). The final dataset used consisted of 152 measurements from 32 subjects with age, height, weight, sweat rate, sweat lactate, heart rate, and ELER as independent variables and blood lactate as the dependent variable. Once the dataset was built, 80% of it was used to perform supervised training of the model, while the remaining 20% is used for testing the prediction capacity of the trained model. Different linear models were evaluated for multiparametric regression such as linear model (LM), partial least-squares (PLS) regression, or principal component regression (PCR). Moreover, a neural network algorithm (multilayer perceptron, MLP) was implemented to take into account the non-linearity and complexity of the data. The metric used for validating the model prediction accuracy was the root-mean-square error (RMSE). More details can be found in the Supporting Information.

## Results and Discussion

### Characterization of the Sweat Lactate Sensor

The lactate sensor used for sweat measurements uses a two-electrode cell functionalized with a membrane of lactate oxidase (LOX) for the amperometric measurement. In this technique, a constant potential, previously determined by cyclic voltammetry, is applied to produce the redox reaction at the electrode surface. The current generated by the electron transfer of the enzymatic reaction depends on the lactic acid concentration of the sample. Although the strips used are intended for capillary blood, the same measurement can be applied in sweat. The two main aspects to be taken into account are the interferents present in sweat (effect of the sample matrix) and the larger concentration of lactic acid found in sweat compared to blood.^13^ The sweat matrix was replicated by using an artificial formulation, and the range of lactic acid was tested beyond the fabricant specifications in blood.

**Figure 2:**
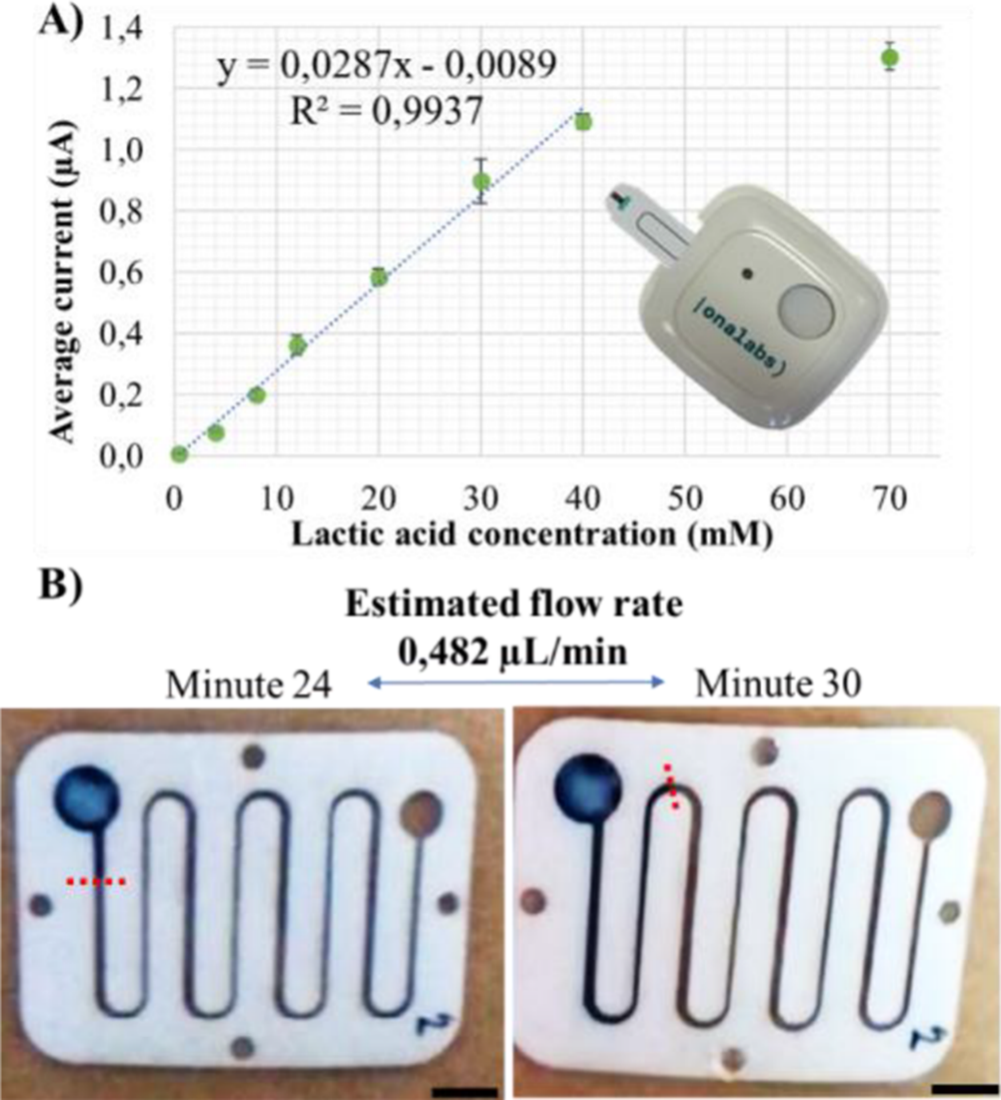
Sweat sensors used in this study. (A) Calibration curve of the sweat lactate sensor in artificial sweat solution. (B) Images of the sweat rate sensor during use in a subject showing the advancing sweat front for a given interval of time, from which the sweat rate is calculated (scale bar = 5 mm).

From the cyclic voltammetry, an oxidation peak was detected at a voltage of 0.055 V corresponding to hydrogen peroxide, a subproduct of lactate oxidation (Figure S1). This low potential of operation is key to reducing the interference from other chemical species present in sweat. The chronoamperometric measurements performed using the potential found in complex samples confirmed that the range of the sensor could be extended up to 40 mM with a good linear response (Figure 2A), and saturation was observed for larger concentrations. The sensitivity found was 0.0287 ± 0.0009 μA/mM. Besides, we demonstrated that the same trend was captured using our developed instrumentation with sufficient resolution (Figure S2).

### Characterization of the Sweat Rate Sensor

The proposed sweat rate sensor determines the sweat volume by continuously monitoring the position of the sweat front in a microfluidic channel with controlled geometrical dimensions. This methodology is based on previous studies that have already been demonstrated in field studies and validated for the local sweat rate measurement against a reference such as the gravimetric measurement using absorbent patches.^31^Figure 2B shows a typical use of the sweat rate sensor attached to skin and how the flow rate is calculated from the volume difference in a given interval of time. The detailed procedure used to calculate the flow rate inside the microfluidic channel using the fluid front position and the geometrical dimensions of the device is included in the Supporting Information (Figure S4).

The device working principle and procedure were validated using a syringe pump, which provided a known flow rate. The syringe pump flow rate error was around 0.4% at 1 μL/min (measured using gravimetric analysis), while the averaged relative error for our sensor was 5.7% (*n* = 3). This deviation is considered low enough to validate the use of our portable sweat rate sensor for in vivo tests (Table S1 and Figure S5). The comparison between the individual device dimensions and the averaged dimensions (obtained from a subset of 10 devices) confirmed the reproducibility of the fabrication method.

### In Vivo Proof of Concept

The methodology proposed for in vivo tests was validated using controlled conditions in a laboratory environment before applying it to the field tests. The sampling patch for the sweat lactate measurement collected enough sample to fill the capture zone in less than 3 min just after cleaning it due to the hydrophilicity of the channel. This feature allows measurements with a frequency of 3 min, higher than the majority of the bioequivalence studies found in the literature.

Preliminary in vivo tests provided meaningful information to improve the performance of the sweat rate sensor. First, the inlet was placed at a certain distance from the edge of the device (more than 8 mm in our case) to provide an intimate contact with skin and prevent leakages that could mislead the results. Besides, the filter paper was placed at the same inlet in order to reduce the dead volume up to the sensing microfluidic channel, minimizing the lag time for the first measurement. Finally, the inlet dimensions that provide the sweat collection area should be designed depending on the total volume of the microfluidic channel. Therefore, considering local sweat rates at the chest zone,^33^ a volume of the microfluidic system of 24.3 μL should provide more than 2 h of working time until saturation (completely filled) for a 4 mm diameter inlet.

### Bioequivalence Analysis

First of all, the high number of tests carried out allowed us to study the variability between the current instrumentation available for the blood lactate measurement. The colorimetric method, stablished as the reference in the laboratory, was compared to the electrochemical portable meter used for field measurements. It was found that the commercial portable meters used in sports medicine had a deviation from reference results of 1.3 mM (RMSE) (Figure S7). Therefore, this degree of variability is accepted in the market for field tests and sets the threshold for the desired performance of our non-invasive system.

As reviewed before, the lactate bioequivalence from sweat to blood is a complex process because there is not a direct correlation. This result was clear in our data as well, as shown in Figure S8, with different phenomena potentially masking a relationship. Therefore, a multiparametric approach must be implemented combining robust sweat-based measurements with additional non-invasive parameters from the user. The first set of models applied were multiparametric regression models: linear, PLS regression, or PCR. The performance obtained with these models was poor in terms of accuracy and robustness (Table S2).

**Figure 3:**
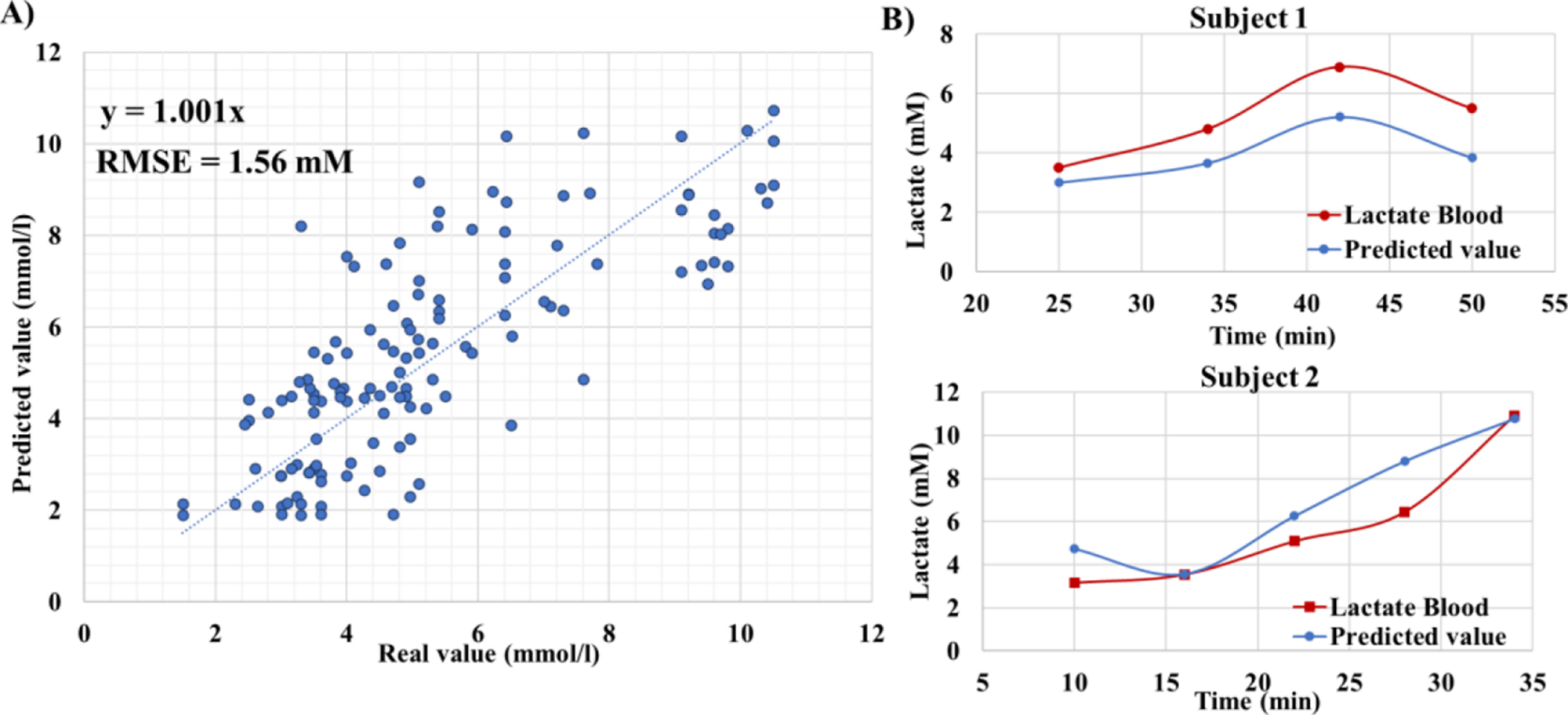
Prediction using the model trained (MLP). (A) Correlation plot between actual values of blood lactate (measured using portable meters) and predicted values using non-invasive parameters and the machine learning model. (B) Evolution for two subjects of blood lactate (actual and predicted) to show the capability of predicting for a particular test.

A neural network algorithm (MLP) was implemented to increase the complexity and introduce the non-linearity into the variable relations. The initial results showed a greater deviation with respect to commercial meters (RMSE = 2.33 mM, Figure S9A). However, when filtering out high blood lactate values, which can distort the model due to their low frequency and presented a significant increase in error prediction, the performance was notably increased (RMSE = 1.56 mM), Figure 3A. The trained model also showed to be able to predict the whole temporal profile in blood lactate of a given subject, whose data were not used for model training (Figure 3B). The relative importance of each independent variable in the prediction of the neural network can be extracted (Figure S9B). ELER resulted in the most important parameter, validating that the initially stablished relation has a significant role in the prediction. Furthermore, the most relevant parameters were all sweat-based plus the heart rate, without relevant contribution from subject metadata.

## Conclusions

The results of this study support the idea that the estimation of blood lactate is feasible using non-invasive sweat measurements, opening the road for continuous, remote, and autonomous monitoring of lactate for sports and health training. The parameters considered were sweat lactate, sweat rate, heart rate, and, with less significant contribution, subject metadata that could be easily obtained such as age, height, and weight. A neural network algorithm was used to predict blood lactate values (RMSE = 1.56 mM), a methodology that can be applied to real situations, with a demonstrated accuracy close to current portable blood lactate meters (RMSE = 1.3 mM), resulting in less than 0.3 mM of accumulated error. Although the methodology proposed has successfully achieved the objective of confirming lactate bioequivalence, several improvements must be implemented in order to provide a wearable lactate monitoring system.

First, at the sensor level, a sweat lactate sensor must be capable of providing continuous measurements for the typical duration of a physical exercise (1–2 h, even longer) while satisfying fabrication and storing conditions for commercial purposes. Then, it must be integrated into a microfluidic system dedicated to the sampling and renewal of sweat, as achieved manually in this work. The same principles must be applied to the sweat rate sensor, where a continuous and automatic measurement method must be used. To obtain these data and process them in real time, the system must incorporate the required electronics in charge of the sensor instrumentation, the data processing, and the remote communication. The sensor instrumentation must be tailored for each specific sensor for integration purposes, while the data processing capability must be enough to embed the required prediction algorithms.

The presented prediction system in combination with a sweat-based platform can be directed to diverse applications, such as dehydration or health-related biomarkers, promoting a great leap toward personal monitoring. However, all future devices must be tailored to the final use case in terms of the parameters measured, sweat sampling, and data analysis. It is certain that the sweat monitoring process is more challenging than the current gold standard, but we believe that application-driven solutions will have a strong impact on sports and healthcare sectors in the near future.

## Supporting Information Available

Sensor characterization, microfluidic construction, methodology, in vivo protocols, data analysis, and prediction models (PDF)
