# Supplementary material for: Non-Invasive Multiparametric Approach To Determine Sweat–Blood Lactate Bioequivalence
Source: ACS Sens. 2023 Apr 8;8(4):1536–41. doi: 10.1021/acssensors.2c02614 (PMC10152482; doi:10.1021/acssensors.2c02614)
Supplement: Supplementary file 1 — se2c02614_si_001.pdf [file se2c02614_si_001.pdf]

Supporting Information

A non-invasive multiparametric approach to determine sweat-blood lactate bioequivalence

Genis Rabost-Garcia[1,2]*, Valeria Colmena[2], Javier Aguilar-Torán[2,3], Joan Vieyra Galí[2], Jaime Punter-Villagrasa[2], Jasmina Casals-Terré[1], Pere Miribel-Catala[3], Xavier Muñoz[2], Joan Cadefau[4], Josep Padullés[5], Daniel Brotons Cuixart[6]

*Corresponding autor: genis.rabost.garcia@upc.edu

[1] Department of Mechanical Engineering (DEM), Universitat Politècnica de Catalunya - BarcelonaTech (UPC), 08222, Terrassa, Spain.
[2] Onalabs Inno-hub S.L., 08290, Cerdanyola del Vallès, Spain.
[3] Electronics and Biomedical Engineering Department, Universitat de Barcelona (UB), 08028 Barcelona, Spain.
[4] Institut Nacional d'Educació Física de Catalunya (INEFC), 08038, Barcelona, Spain.
[5] Associació Chronojump, 08013, Barcelona, Spain.
[6] Consell Català de l'Esport, 08950, Esplugues de Llobregat, Spain.

Sweat Lactate Sensor: Characterization and Instrumentation

Lactate Pro-2 test strips (Akray, Kyoto, Japan) were used as single-use sweat lactate sensor. As detailed in their datasheet, they contain an enzymatic membrane made of Lactate Oxidase (2 IU) and mediators such as Hexaammineruthenium (III) chloride and 1-Methoxy-5-methylphenazinium methylsulfate in order to perform amperometric measurements in capillary whole blood. The test strip includes a fluidic chamber of 0.3 µL which absorbs the sample spontaneously by capillarity and keeps constant the volume of sample to be measured. In conjunction with their dedicated reader, the test strip is able to provide a linear range from 0.5 to 25 mM in capillary whole blood.

Figure S1 shows the in vitro characterization carried out for the adaptation of the test strip to sweat measurement. First, Figure S1A shows the results obtained from a cyclic voltammetry (from -0.1 to 0.8 V at a scan rate of 0.02 V/s) of a 10 mM lactic acid solution in 0.1 M phosphate buffer at pH 6.3. This way, the operating potential of the redox reaction of interest is defined. At that given potential, chronoamperometries are performed for increasing lactic acid concentrations in artificial sweat (phosphate buffer (pH = 6.5), 50 mM NaCl, 0.17 mM glucose, 5 mM NH_4Cl, 20 mM urea, 0.03 mM ascorbic acid) to check sensor response (Figure S1B).

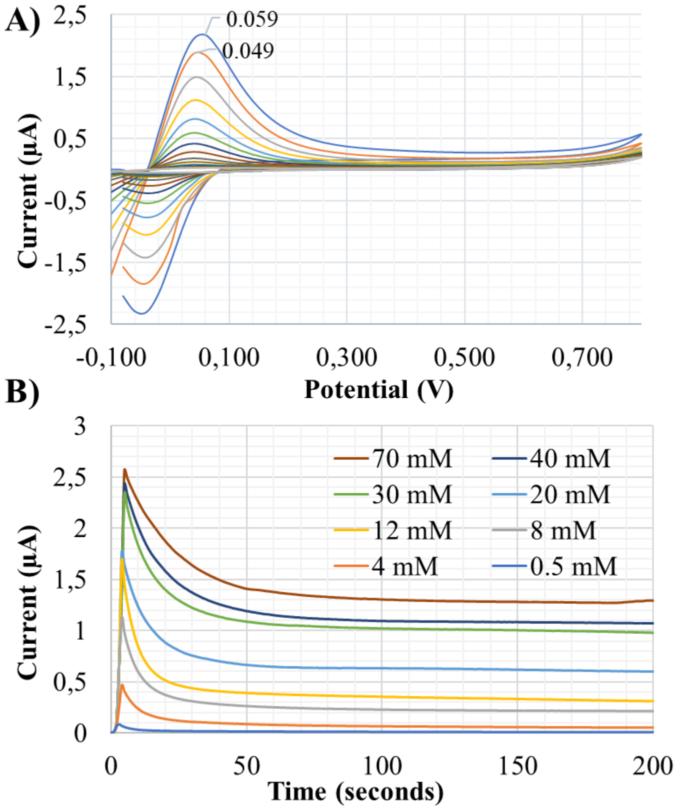

Figure S1. Electrochemical characterization of the Lactate Pro-2 test strip using reference instrumentation. A) Cyclic voltammetry (15 cycles to check system stability) in a solution of 0.1 M phosphate buffer pH=6.3 and 10 mM of lactic acid. B) Chronoamperometries at different concentrations of lactic acid in artificial sweat matrix (0.05 V applied for 200 sec).

The results from the chronoamperometries were used to design a custom potentiostat to perform this measurement of sweat lactate sensor. This custom reader is key for field studies since it provides real-time wireless measurements, therefore it simplifies the measurement process and allows to know that sample has been captured correctly by the test strip. The minimum current to be measured is 8 nA for the 0.5 mM lactic acid concentration (a rare case in *in vivo* tests), but the resolution of the developed potentiostat is ±6 nA[1]. Figure S2 shows a comparison of the results obtained with the new instrumentation and the ones obtained with the reference instrumentation (Palmsens 4, Palmsens BV, Netherlands).

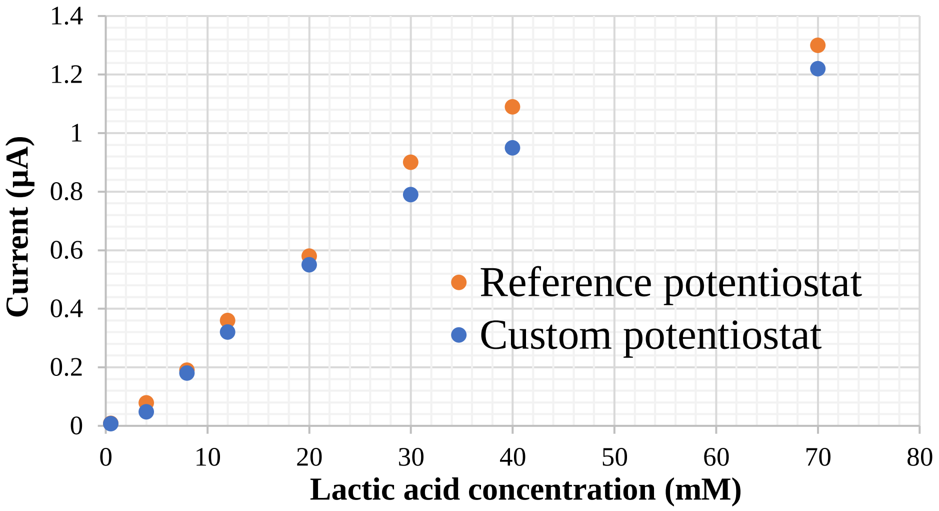

Figure S2. Comparison of the readings of the instrumentation. Current measurement for the whole range of lactic acid concentrations for the reference potentiostat (orange) and the custom potentiostat (blue).

Epidermal patches Construction: Sampling Patch and Sweat Rate sensor

The sampling patch is a three-layered device that delivers the sweat samples to the sweat lactate sensor. The initial layer (ARcare 90445) interacts with skin and defines a high area of recollection (1.95 cm^2) to allow enough sweat volume to perform repeated measurements with a frequency under three minutes, if required. Then, the collected sweat is canalized using hydrophilic adhesives (ARflow 93049) for spontaneous, fast filling up to the capture zone. A plastic spacer of PMMA is placed to define the capture zone and facilitate test strip allocation and sweat capture during *in vivo* tests. Figure S3A is an exploded view of the sampling patch.

In a similar manner, a three-layered laminated device is built for the sweat rate measurements. The first layer (ARcare 90445) interacts with skin and defines the inlet (4 mm diameter, 12.6 mm^2) from where sweat is collected into the microfluidic channel. The microfluidic channel is defined in a hydrophobic adhesive (second layer, ARcare 90106) using laser cutter, while the height is the thickness of the layer (0.193 mm according to fabricant). The serpentine format allows for a higher volume to area ratio, increasing the time of use of the device. The layer 3 (ARcare 90445) serves as a cover of the microfluidic channel and outlet. The filter paper (Whatman 50) used was selected because its reduced thickness (0.12 mm) provided a better integration inside the microfluidic device. A circle of 5 mm diameter was punched out and placed before laminating layer 1. Figure S3B is an exploded view of the sweat rate sensor.

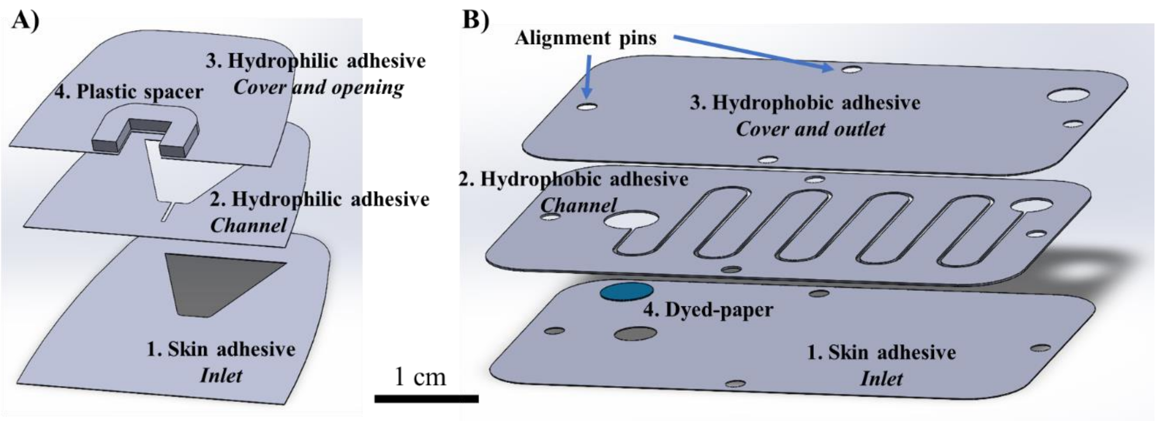

Figure S3. Exploded views of the epidermal patches used in the study. A) Sampling patch for sweat lactate measurement. B) Sweat Rate sensor.

Sweat Rate Sensor: Characterization

The procedure to calculate the flow rate in the microfluidic channel starts with the determination of the sweat front relative to the correspondent section of the microfluidic channel (Figure S4). Image J software was used to measure the filled part length in pixels compared to the whole-section length, obtaining a filled percentage of that specific section. The previous sections are considered fully filled (filled percentage = 1) while sections after are considered fully empty (filled percentage = 0). Knowing the geometrical dimensions of each section, the filled volume corresponds to the geometrical volume multiplied by the filled percentage and the total volume is the sum across all sections. Once volume at a given time point is calculated, sweat rate is found by the difference in volume between two time points.

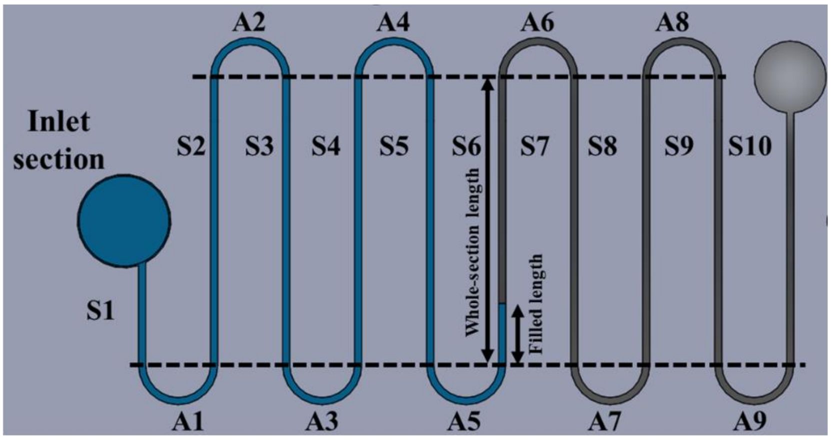

$$Filled\ percentage\ (FP) = \begin{Bmatrix} Inlet, S1, A1, S2, A2, S3, A3, S4, A4, S5, A5 = 1\ (Fully - filled) \\ S6 = \frac{Filled\ length\ (px)}{Whole{-}section\ length\ (px)}\ (Filling) \\ A6, S7, A7, S8, A8, S9, A9, S10 = 0\ (Fully - empty) \end{Bmatrix}$$

$$V_{inlet} = \underbrace{A_{inlet}\ x\ H_{layer1}}_{V_{layer1}} + \underbrace{A_{inlet}\ x\ H_{layer2}}_{V_{layer2}} - \underbrace{(1-\Phi)\ x\ H_{paper}\ x\ A_{paper}}_{V_{paper}}$$

$$V_{section} = L_{section}\ x\ W_{section}\ x\ H_{layer2}$$

$$\boxed{Volume\ (\mu L) = \sum_{Inlet\ section}^{S10} (V_{section}\ x\ FP_{section})}$$

Figure S4. Procedure to calculate sweat rate, scheme of the sweat rate sensor and equations implemented.

The volume of the inlet section was also estimated in order to fasten the response of the sensor (Figure S4). With the system presented, no measurement can be done until sweat has arrived to the main channel and the first capture serves only as a basal volume for next measurement. However, if inlet volume and starting time is known, an approximated sweat rate can be estimated from the first measurement. Calculation of this volume includes the volume of the layer 1 and the volume of the layer 2 minus the volume occupied by the paper, which results in approximately 3.5 µL.

The geometrical dimensions of the microfluidic channel were validated in an initial batch of devices to provide average values to be used. The in-plane dimensions (defined by laser cutting) showed variability across channels due to the fabrication technique but the impact on the calculated flow rates was minimal. The height measured using an optical profilometer confirmed the manufacturer thickness. The calibration process was done against the flow rate provided by a calibrated syringe pump. This methodology verified the operation, the sensitivity required and the sweat rate calculation procedure.

Table S1. In vitro characterization of the sweat rate sensor. Values in parenthesis are obtained using dimensions of that individual device, the others are obtained from the averaged values.

	Averaged sweat rate (µL/min)	Relative error (%)
Syringe pump (gravimetry)	0.998	0.4
Sweat rate sensor #1	0.954 (0.959)	6 (7)
Sweat rate sensor #2	0.931 (0.971)	7 (6.5)
Sweat rate sensor #3	0.965 (0.959)	4 (7)

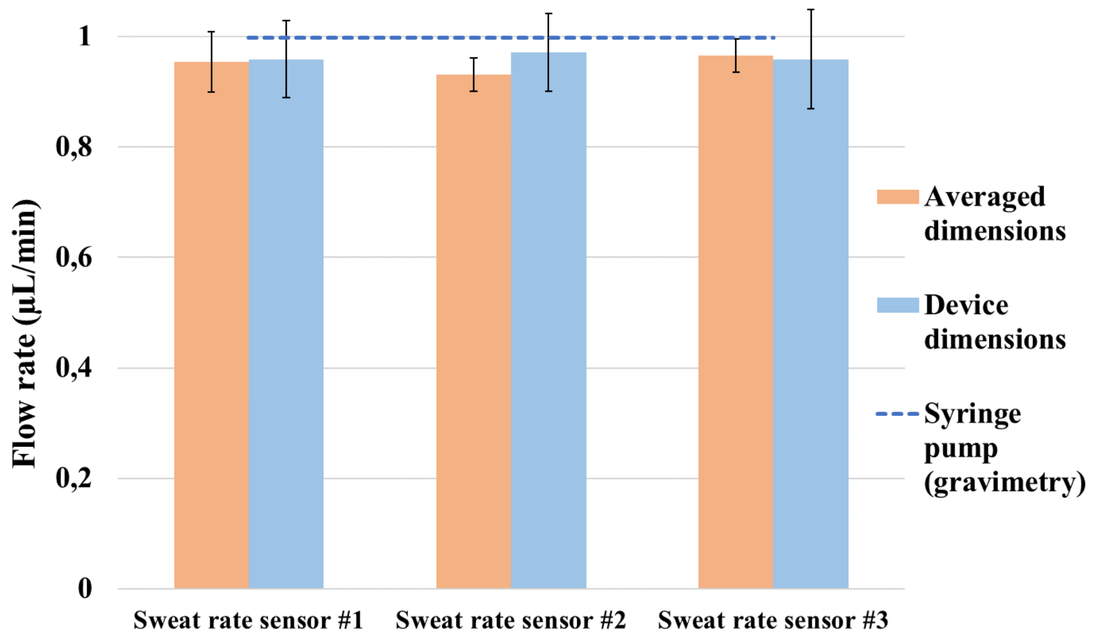

Figure S5. In vitro characterization of the sweat rate sensor using a syringe pump at a constant flow rate. The response of the syringe pump has been calibrated using gravimetric measurements.

In vivo studies

<u>Methodology</u>

The method consists of the following steps (Figure S6):

1) Skin cleaning prior to the attachment of the sampling patches is key to ensure a good adhesion with the sampling patches, by removing any contaminant or skin-care product present[2]. First, ethanol was applied using sterile gauzes at the zone of application (chest area). Then, a DI water-soaked sterile gauze was used to remove completely ethanol which may prevent sweating. Finally, skin was wiped dry with a new sterile gauze before the attachment of the sampling patches on the chest of the subject. The heart rate strap monitor was also placed before starting the test.
2) Starting the test which can be at a cycloergometer or running track. More details on the tests carried out below.
3) Once the subject started sweating, usually around 10-15 minutes into the test, simultaneous measurements of blood lactate, sweat lactate, sweat rate and heart rate were performed.
4) For blood lactate, a puncture was done at the earlobe to draw blood to perform the measurement. Measurements were done using Lactate Pro2 meter and test strips (Akray, Japan) and also Lactate Plus meter and their test strips (Nova Biomedical, USA). For some subjects, blood was collected in a capillar for later testing with a reference instrumentation at laboratory (colorimetric photometer, Diaglobal, Germany).
5) For sweat lactate, a Lactate Pro2 test strip was placed into the custom reader and the mobile app started to receive the measured signal. The test strip is approached to the capture zone of the sampling patch and when sweat enters to the sensing chamber a peak appears on the measurement screen due to the start of lactate oxidation. Chronoamperometry data can be saved for later analysis with time traceability. The capture zone of the sampling patch was cleaned using cotton swabs of the excess sweat. The test strip was discarded and a new one was used for the next measurement.
6) For the sweat rate measurement, a picture was taken with the smartphone ensuring that the fluid front was distinguishable (it has been noted that flash allows for better contrast). Image was saved for later analysis with time traceability.
7) For the heart rate measurement, the heart rate strap monitor data was transferred to their own mobile app which can be accessed after the test for analysis with time traceability.
8) The process was repeated for each measurement for a given subject until the test was finished with variated frequency depending on the test. Typically, there were from 4 to 8 measurements for subject.

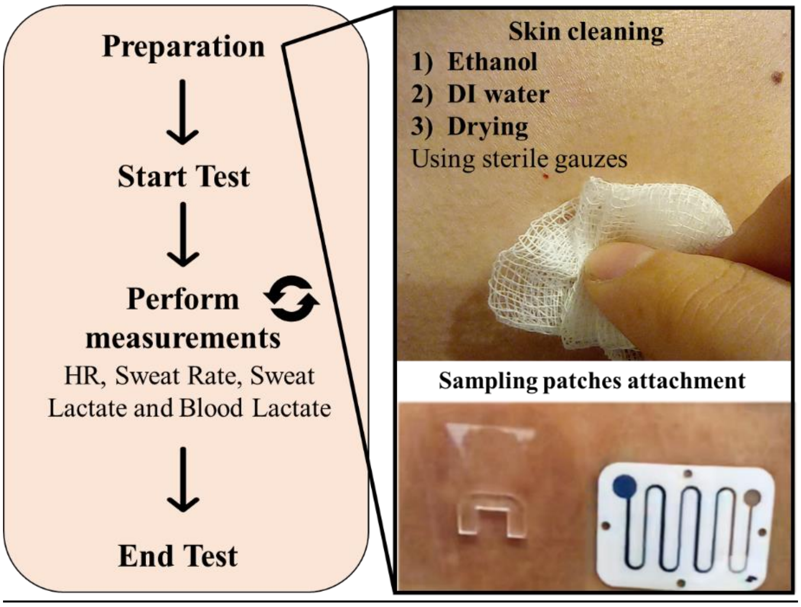

Figure S6. Scheme showing the measurement procedure, focusing on skin preparation for sampling patch attachment, for in vivo tests.

Field studies

Variated physical tests were carried out with the goal of obtaining a wide range of lactate concentrations, subjects for a complete bioequivalence study. Tests were carried out with volunteers, athletes of the Centre d'Alt Rendiment (CAR) and students of the Institut Nacional d'Educació Física (INEFC) from February 2021 to April 2021. The dataset contained 152 measurements from 32 subjects. All subjects of the study were young adults due to the high percentage of athletes inside this age group and availability restrictions. In terms of gender and training level, there was enough variability, but a distinctive analysis was not carried out as the main objective was absolute bioequivalence. The protocols carried out were intended to mimic the actual tests and trainings carried out by the athletes or volunteers for blood lactate testing. Apart from the setting (cycloergometer or running track), we differentiated in terms of intensity between incremental and constant load. The duration of the test were matched with the typical ergometry tests carried out in sports medicine. It was relevant to mix both types of activity because they produce different responses in lactate levels, increasing the variability of the data and making the posterior analysis more robust.

Cycloergometer test – Incremental intensity

- 6 subjects, N = 38 measurements, $N_{average}$= 6.33 measurements/subject.
- Warm-up for 10 minutes at 50-100 W power load.
- After warming up, increase of power 30 W for 3 minutes.
- Measurements performed after each period of 3 minutes.
- Increasing up to subject maximum capacity or cycloergometer capacity.
- After maximum reached, 3-minute period of active rest followed by 3-minute period of complete rest. Measurements after each of these periods as well.

Running track test – Constant intensity

- 22 subjects, N = 92 measurements , $N_{average}$= 4.18 measurements/subject.
- Subjects already warmed up.
- Four series of 1200-2000 meters.
- Measurements performed after each series and after resting for 3 minutes. Time between measurements depended on the subject pace but close to 6 minutes.

Triathlon (Cycloergometer-Running Track) test

- 4 subjects, N = 21 measurements, $N_{average}$= 5.25 measurements/subject.
- Subjects already warmed up.
- Cycling for 10 minutes followed by 400 meters running track. Three consecutive series and measurements were performed after each transition and after resting for 3 minutes. Time between measurements depended on the subject pace but close to 10 minutes.

Data Analysis

The analysis process started by extracting data from the sensors. For the sweat lactate measurements, the averaged current of the stable region of the chronoamperometry was used directly, as its proportionality with lactic acid concentration had been previously demonstrated (Figure 2A, S1B). For the sweat rate measurements, the procedure described for *in vitro* characterization was applied. The heart rate was extracted from the heart rate monitor source for the same test time as the rest of measurements. The lactate Excretion Rate (LER) is the product of the sweat lactate concentration and the sweat rate to account for volume dilution. The LER was divided by the heart rate (HR) to create the ELER (Exertion and Lactate Excretion Rate) parameter as a way to introduce the expected relation between independent variables. The purpose the ELER parameter is to provide the models with an initial logical relation between independent variables. On the contrary, if the relation is not present in the data, model performance would be reduced.

Pre-processing for all models used consisted in centering and scaling for all continuous variable in order to have a mean of 0 and a standard deviation 1. This way, the numerical stability is improved and continuous variables of different values can be used together. The initial set of models used for the prediction of blood lactate are multiparametric linear models including LM (Linear Models), PLS (Partial Least Squares) or PCR (Principal Component Regression). Due to the complexity of the data, it is expected that non-linearity must be included through the use of a neural network algorithm (MLP, MultiLayer Perceptron).

The structure of MLP consists in an input layer (independent variables), a hidden layer and the output layer (dependent variable). MLP just have a single hidden layer with neurons (hidden units) which are mathematical expressions consisting of weighted inputs (obtained from supervised back-propagation training) that produce an output only above a certain threshold. This threshold is controlled by the activation function (which can be of different nature) which is the non-linearity term added to the model. MLP used is from the package *caret* in R, the number of neurons in the hidden layer was tuned (from 1 to 10, 5 neurons were the final optimized number) and the activation function used was the rectified linear function.

The statistical metrics used to test the accuracy of prediction of the model was RMSE. RMSE (Root Mean Square Error) is the square root of the squared differences between real (blood lactate values) and predicted (from the trained model) values and provides information about the inaccuracy of the prediction in the same units as the variables which facilitates comprehension.

Blood Lactate measurement variability

As we measured the same blood sample with the two measurement systems available currently for lactate determination (the colorimetric photometer at laboratory, Diaglobal, and the electrochemical portable meters, Lactate Plus), the variability associated to the field methodology can be studied. There were enough measurements to carry out the cross-correlation, shown in Figure S7A. It was found a significant deviation between the two methodologies of 1.3 mM (RMSE), but this error is accepted by the sports field and it is the standard degree of accuracy required for lactate monitoring tool. Figure S7B is a Bland-Altman plot, difference between both methods across the range of measurement, with the 95% (±1.96 SD) confidence intervals for better assessment of the degree of agreement.

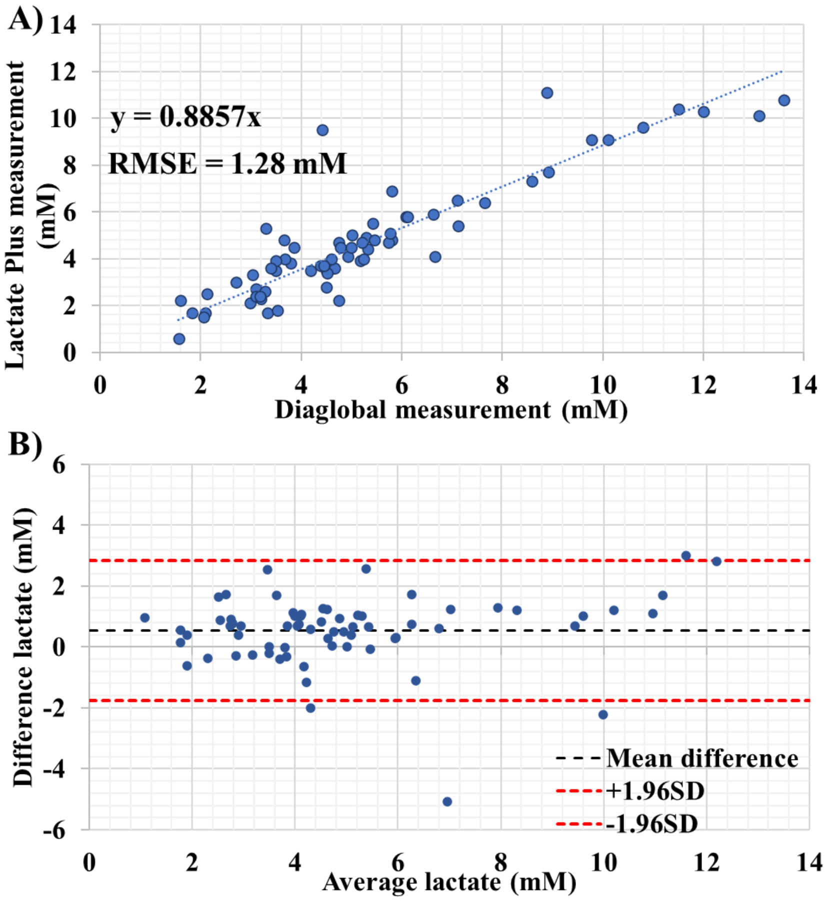

Figure S7. A) Correlation plot between Lactate Plus (portable meter and test strips) and Diaglobal (optical instrument) for blood lactate. B) Bland-Altman plot showing the agreement between methodologies for blood lactate sensing (colorimetric procedure versus electrochemical meters).

Bioequivalence results

Once the dataset was built, the first step consisted in finding the relationship between blood lactate and sweat lactate. A simple correlation plot was enough to see that direct correlation was not feasible, see Figure S8, and that multiparametric approaches must be used.

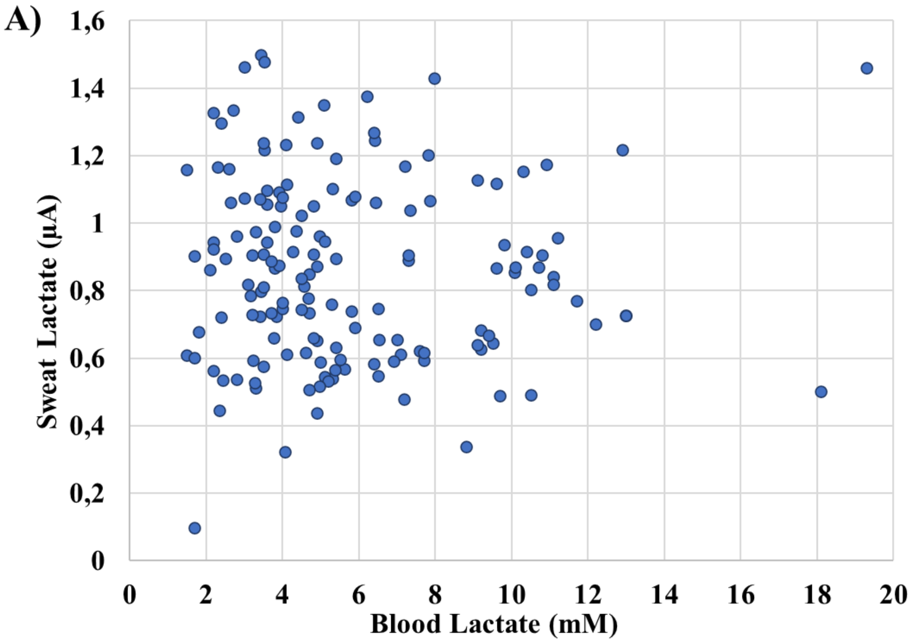

Figure S8. Graph showing the relation of blood lactate versus sweat lactate values.

The first set of models applied were multiparametric regression models: PCR, PLS and LM. All these algorithms are linear, therefore allowing for simpler, less computing intensive predictions. However, the performance obtained initially was not satisfactory enough to use them as a reliable tool for the blood lactate prediction. Not only in the accuracy (reflected by the RMSE value), but the prediction robustness was not reproducible at all, depending greatly on the training data used. On the other hand, MLP showed a robust prediction with minimal variation to training data, as well as an increase in prediction accuracy.

Table S2. RMSE values of the models used.

**Model**		**RMSE (mM)**
**PCR**	*Principal Components Regression*	2.78
**PLS**	*Partial Least Squares*	2.72
**LM**	*Linear model*	2.60
**MLP**	*Multi-layer perceptron*	2.33
**MLP**	*Multi-layer perceptron (BL < 10.5 mM)*	1.56

A correlation plot of multiple predictions combined using MLP algorithm is shown in Figure S9A. It can be clearly seen how high blood lactate values are scarce in our dataset and have the least accurate predictions. Besides, the relevance in predicting high values of lactate is limited as the athlete would be far past the anaerobic threshold. RMSE, the metric used for accuracy assessment, was calculated for different intervals and values over 10.5 mM were discarded due to a significant increase in the prediction error compared with the rest of observations.

A meaningful characteristic of the MLP model used is the relative importance of each independent variable in the prediction (Figure S9B). The most important parameter was found to be ELER, the derived parameter from the rest of measurements, confirming that the relationship envisioned was correct. The next variables, in terms of importance, were the individual measurements of heart rate, sweat lactate and sweat rate. These results support the idea that the non-invasive measurements carried out are the basis of the lactate bioequivalence results showed in this work, and there are not confounding factors such as subject characteristics or testing practices.

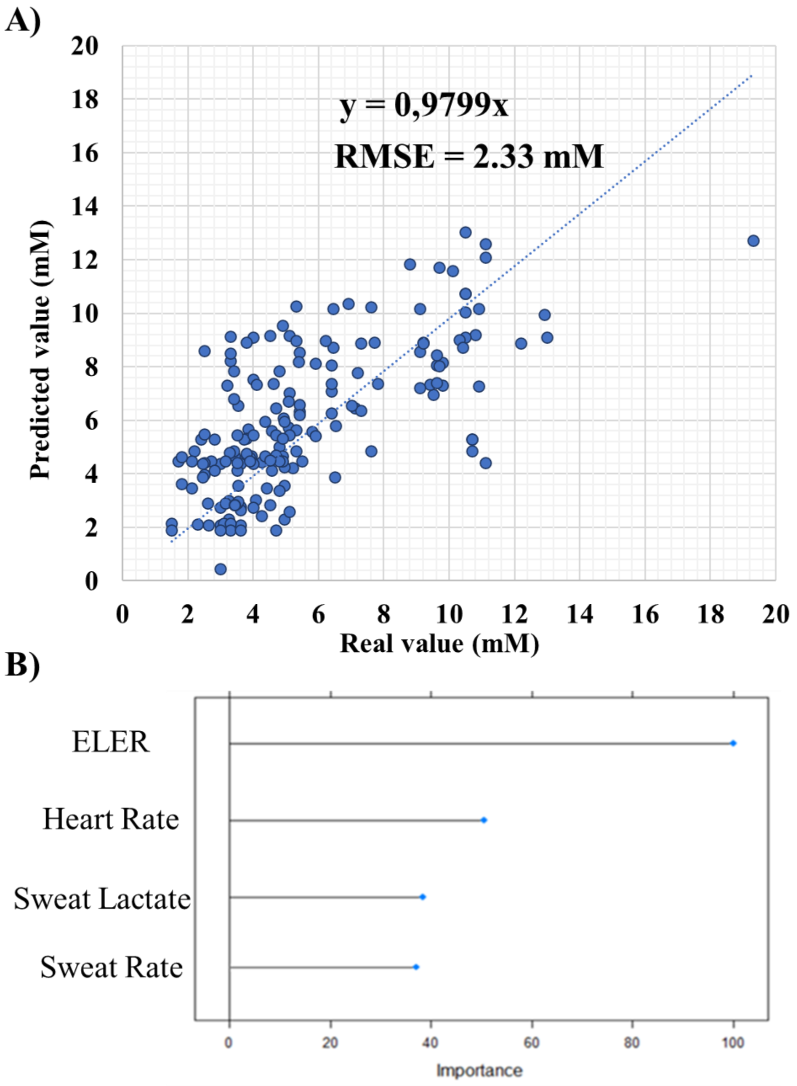

Figure S9. Additional information to the prediction using MLP model. A) Correlation plot with complete data, without filtering out high blood lactate values (>10.5 mM). B) For the selected model (MLP), importance inside the model of each input parameter.

References

1. Aguilar, J.; Alvarez-Carulla, A.; Colmena, V.; Carreras, O.; Rabost, G.; Puig-Vidal, M.; Colomer-Farrarons, J.; Munoz, X.; Miribel-Catala, P.; Punter-Villagrasa, J. Autonomous Self-Powered Potentiostat Architecture for Biomedical Wearable Applications. In *2020 35th Conference on Design of Circuits and Integrated Systems, DCIS 2020*; Institute of Electrical and Electronics Engineers Inc., 2020.

2. Baker, L. B. . Sweating Rate and Sweat Sodium Concentration in Athletes: A Review of Methodology and Intra/Interindividual Variability. *Sports Medicine*, *47*, 111–128, 2017.
